# Having a direct look: Analysis of DNA damage and repair mechanisms by next generation sequencing

**DOI:** 10.1016/j.yexcr.2014.08.011

**Published:** 2014-11-15

**Authors:** Bettina Meier, Anton Gartner

**Affiliations:** Centre for Gene Regulation and Expression, University of Dundee, Dundee, UK

**Keywords:** *C. elegans*, Mutagenesis, Whole-genome sequencing, Mutation profiles, DNA repair pathway analysis

## Abstract

Genetic information is under constant attack from endogenous and exogenous sources, and the use of model organisms has provided important frameworks to understand how genome stability is maintained and how various DNA lesions are repaired. The advance of high throughput next generation sequencing (NGS) provides new inroads for investigating mechanisms needed for genome maintenance. These emerging studies, which aim to link genetic toxicology and mechanistic analyses of DNA repair processes *in vivo*, rely on defining mutational signatures caused by faulty replication, endogenous DNA damaging metabolites, or exogenously applied genotoxins; the analysis of their nature, their frequency and distribution. In contrast to classical studies, where DNA repair deficiency is assessed by reduced cellular survival, the localization of DNA repair factors and their interdependence as well as limited analysis of single locus reporter assays, NGS based approaches reveal the direct, quantal imprint of mutagenesis genome-wide, at the DNA sequence level. As we will show, such investigations require the analysis of DNA derived from single genotoxin treated cells, or DNA from cell populations regularly passaged through single cell bottlenecks when naturally occurring mutation accumulation is investigated. We will argue that the life cycle of the nematode *Caenorhabditis elegans*, its genetic malleability combined with whole genome sequencing provides an exciting model system to conduct such analysis.

The use of next generation sequencing allows for defining the direct consequences of mutagenesis, occurring in response to DNA alterations, such as DNA adducts, single- and double-strand breaks or misincorporation of nucleotides, all modulated by the cellular DNA repair capacity. These emerging studies are based on a long tradition of using mutagens in model organisms, and mutagenesis induced by a variety of agents has formed the basis for unbiased forward genetic screen in yeasts, fruit fly and *C. elegans*. Indeed, in 1927 Müller was the first to find that the exposure of *Drosophila* germ cells to ionizing irradiation (IR) leads to increased mutagenesis [1]. Measuring mutagenesis in *C. elegans* is facilitated by its life cycle. Worms, which reproduce every 3 to 4 days, are hermaphrodites, containing both male and female germ cells. Germ cell nuclei can be treated with mutagens, and gametes derived from these cells fuse to form the zygote. Development into a self-fertilizing adult enables clonal amplification of any quantal DNA damage that was fixed before the zygote divided (see Fig. 1B). Whole genome sequencing of DNA derived from the clonal progeny of single worms allows for detecting mutations, their signatures and distribution. Given that *C. elegans* is diploid, individual mutations are expected to occur in ~50% of sequencing reads in the first filial (F1) generation. Working in a diploid system facilitates survival even with a massive load of heterozygous mutations. In comparison, classic studies rely on scoring the number of mutations leading to visible phenotypes, in the F1 for dominant traits, but more commonly in the F2 generation when recessive traits become visible. The measurement of mutagenesis based on visible mutant counts or by direct next generation sequencing contrasts with toxicology-based approaches, where genotoxin treatment leads to reduced germ cell proliferation and or a reduced rate of progeny survival. Importantly, reduced survival does not necessarily correlate with DNA repair capacity especially when error prone repair modalities are used.

When the rate of mutagenesis is low, for instance when analyzing spontaneous mutagenesis, this is assessing the pace of evolution; worms have to be propagated for many generations, typically 20 or more, to obtain a sufficient number of mutations. *C. elegans* being hermaphrodites allows for propagation of clonal lines, the zygote of each generation being the single cell bottleneck. Sequencing DNA from the initial parental line and the last generation, we now know the baseline mutation rate of ~1 mutation per generation in *C. elegans* [2]. This is ~3 times higher than previously reported, and corresponds to ~6.7×10^−10^ per nucleotide per cell division. This is in line with a reported mutation rate of 3.6×10^−9^ substitutions per nucleotide per generation in *Saccharomyces cerevisiae* [3]. In humans, *de novo* mutation rates are dependent on the fathers age at conception and have been determined as on average 1.20×10^−8^ per nucleotide per generation for 30 year old fathers [4]. Using previous estimates of ~400 germ cell divisions per generation of for a 30 year old male and ~30 for females [5], the average mutation rate is 0.45×10^−10^ per nucleotide per germ cell division for the male lineage.

Mutagenesis is a driving force in tumor progression and both genetic and environmental factors have been identified to increase cancer risk. Sequencing of tumor types of varying origin confirmed a substantial variation in mutation load and mutation profile, reflective of different cellular origin, environmental exposures or DNA repair deficiencies [6–8]. Indeed, 21 different mutagenic imprints or mutational signatures, all causing distinct single nucleotide changes in a preferred sequence context were recently extracted using a large number of mutations from over 7000 cancer samples [9]. While some of these mutational signatures could be assigned to known features of environmental contributors such as CC>TT dinucleotide substitutions associated with UV light exposure, predominant C>A mutations with a transcriptional strand bias suggesting the formation of bulky adducts on guanine residues indicative of tobacco smoke exposure, or even signatures of chemotherapy treatment such as alkylating damage in temozolomide-treated cancers, the etiology of many base substitution signatures is currently unknown [9]. We know less about causes and events leading to global genome rearrangements, but discrete signatures are emerging. Chromothripsis refers to massive localized rearrangements, apparently caused by the fragmentation of a chromosome and the subsequent random assembly with copy number gain and loss of individual chromosomal fragments [10]. In contrast chromoanasynthesis is defined by clustered rearrangements associated with copy number gain and is likely caused by replication fork stalling and template switching [11,12].

The occurrence of mutational signatures upon *C. elegans* mutagenesis analogous to those observed in tumor cells, paved the idea of systematically treating worms with genotoxic agents and analyzing the frequency and nature of resulting mutational signatures. Sequencing EMS, ENU and UV/TMP induced mutations for three decades and extending mutagenesis to a massive scale to isolate tens of mutants in each gene has provided an in-depth picture of mutation load and signature. The strong propensity of EMS to alkylate guanine residues causes a mutation bias of G/C to A/T transitions, which leads to a higher incidence of stop codons, thereby increasing the frequency of loss-of-function alleles [13,14]. In contrast, ENU can modify any of the four nucleotides with some preference of G/C to A/T transitions and codons are equally mutable, so non-null alleles are often generated [14,15]. UV/TMP induces base substitution with lower but equal frequency among all bases; however it preferentially generates small deletion of one to three kilobases [14]. In all cases the occurrence of large deletions or complex chromosomal rearrangements has not been systematically investigated by interrogating whole genome datasets. Treating wild-type and DNA repair defective mutants with known DNA damaging agents provided a proof of concept for this approach, and allowed to determine how DNA repair pathways prevent mutagenesis and contribute to distinct mutational signatures. Treatment with aflatoxin B1, a potent liver carcinogen, leads to a dose dependent increase in single base substitutions, with a preponderance for G>T and G>A mutations in *C. elegans* analogous to the mutational profile found when mutated p53 was sequenced from cancer patients with previous aflatoxin B1 exposure [2,16]. Toxin-induced mutagenesis was observed with very low doses of aflatoxin B1, not sufficient to reduce progeny survival, indicating the enormous sensitivity of this approach. Mutation rates were increased in *C. elegans* lines with nucleotide excision repair deficiency, consistent with the role of this pathway in repairing bulky base lesions [2]. Similarly, cisplatin treatment, an agent that leads to monoadducts but also intrastrand and interstrand cross-links, leads to a high rate of C>A mutations in a CpC context [2]. These mutations increase in frequency with nucleotide excision repair deficiency, but not with DNA cross-link or double-strand break repair deficiency, consistent with being the consequence of cisplatin monoadduct formation.

Propagating *C. elegans* wild-type and DNA repair mutants over many generations without exposure to genotoxic agents allows for assessing which pathways prevent spontaneous mutagenesis associated with replication failure and endogenous mutagens (Fig. 1A). The goal of these models is to recapitulate the mutational signatures observed in cancers, and to, by employing a comprehensive panel of DNA repair mutants, understand how the cellular repair machinery prevents and at times modulates mutagenesis. To determine the evolutionary mutation rate and to address the contribution of different DNA repair pathways to genome stability, a set of gene deletion lines in components of the nucleotide excision repair (*xpa-1/XPA*), base excision repair (*ung-1/UNG*), telomere replication (*trt-1/TERT, mrt-2/RAD1*), apoptosis (*ced-4/APAF1, cep-1/p53*), DNA cross-link repair (*fcd-2/FANCD2*) and non-homologous end joining (*lig-4/LIG4*) pathways were grown for 20 generations [2]. Five lines were propagated in parallel for each genotype and randomly selected, individual worms were transferred as a single cell bottleneck each generation. Interestingly, mutation rates were largely unaltered across genetic backgrounds over the course of the experiment suggesting robust cellular DNA repair even when one of several DNA repair pathways was impaired. A previous study, using sequence analysis across multiple genomic loci in mismatch repair mutants (*msh-2/MSH2, msh-6/MSH6*), nucleotide excision repair (*xpa-1/XPA*) and base excision repair (*nth-1/NTH*) mutants indicated a relative importance of MMR>NER>BER in genome maintenance under normal growth conditions [17,18]. While an *xpa-1*-dependent increase in mutation rate and the relative importance of NER *versus* BER was not observed by whole-genome analysis, the importance of the mismatch repair pathway in correcting replication errors and polymerase slippage, leading up to a 100 fold increase in mutation rates has been corroborated in all systems studied [3,19–21]. In the case of base excision repair (BER), the effect on genome stability might vary between different gene deletions due to pathway redundancies, thus requiring further detailed analysis.

The analysis of lesions associated with defects in the DOG-1/FANCJ helicase provides a most elegant use of *C. elegans* classical genetics and NGS sequencing to analyze how combinations of repair factors affect mutagenesis. DOG-1 was initially uncovered as a *C. elegans* mutation that causes an increased frequency of mutations. In a first seminal paper *Cheung et al* found that such mutations preferably affect loci of G-rich sequences with the potential to form G-quadruplexes (G4) [22]. In G4 structures guanines stack into stable, four-stranded secondary structures. Such structures can impede replication fork progression and are prone to induce deletions. In *dog-1* mutants, most deletions were in the range of 50 to 300 bp [22,23]. Functional conservation has been shown for human FANCJ; *in vitro* studies confirmed FancJ DNA helicase activity in resolving G4 DNA sequences and a bias towards deletions in G-rich DNA regions has been observed in FA-J patient cell lines [24,25]. Inactivation of several DNA repair genes, including *him-6*/BLM, *xpf-1/XPF* or *rad-51/RAD51* in a *dog-1* mutant background increased the deletion frequency but did not alter deletion sizes and breakpoint sequences as observed by sequencing of a small number of deletion products upon PCR amplification [26]. Using a more powerful reporter system that allowed for visual selection of deletions at recombinant G4 DNA sequences stably integrated into the genome, Koole et al. were able to isolate over 100 *dog-1*-induced deletions [23]. Deletions were remarkably uniform in size distribution, ranging from 50 to 300 bp and the breakpoints of deletions, which were not associated with additional, inserted DNA sequences, contained minimal breakpoint sequence homology, largely comprised of a single nucleotide [23]. Similar deletions became evident throughout the genome upon NGS sequencing of *dog-1* mutants propagated for 50 generations and, albeit not focused on any discernable sequence motive, accumulate in *polh-1; polk-1* double mutant worms [19,23]. *C. elegans* POLH-1 and POLK-1 are homologs of the human DNA polymerase eta/POLH and kappa/POLK, respectively, error prone translesion synthesis polymerases that read past damaged bases. Intriguingly, deletion of *polq-1*, the *C. elegans* polymerase theta/*POLQ* homologue, in *dog-1* single and *polh-1; polk-1,* double mutants leads to much larger >5 kbp deletions and the absence of any microhomology at the breakpoint [19,23]. These results indicate a function of polymerase theta/*POLQ* in the repair of genomic DNA lesions arising at genomic sites such as G-rich sequences, generating an imprint of DNA repair of 50 to 300 bp deletions. The presence of single base nucleotide homology at the breakpoint suggests POLQ might stabilize structures, where upon DNA double-strand processing, resected 3′ prime single-strand overhangs base pair at their complementary terminal nucleotide (Fig. 2A). Annealing is likely followed by DNA synthesis and DNA ends are ligated in an alternative end-joining pathway independent of non-homologous end-joining proteins (Fig. 2A). Interestingly, some *polq-1*-dependent deletions contain insertions of DNA sequences “templated” from adjacent DNA regions, supporting the notion of a replication-associated mechanism [19,23]. These *C. elegans* studies provide mechanistic insights into POLQ function and are relevant to studies in mammalian cells, where a polq deletion induces IR and bleomycin hypersensitivity and spontaneous micronuclei formation, an indicator of unrepaired DNA damage during anaphase [27–29]. Similarly overexpression of POLQ is associated with reduced replication fork speed and spontaneous chromosomal instability in mammalian cells [30,31]. Thus, POLQ expression likely requires tight regulation to ensure genome stability.

Chromothripsis, a complex type of chromosomal rearrangements, was recently described to occur in 2–3% of tumor samples and to be enriched in bone marrow tumors [10]. Chromothripsis (“chromo” for chromosome and “thripsis” for shattering) is characterized by often massive, highly localized rearrangements, likely caused by a shattering of a chromosomal region or chromosome followed by DNA fragment ligation in a one-off event [10], challenging the long-standing idea of progressive mutation accumulation during cancer development. Telomere attrition has been discussed as one of the possible mechanisms leading to chromothripsis, giving rise to fused chromosomes, which can experience massive DNA breakage at the cleavage furrow during cytokinesis. Interestingly, we found that *C. elegans mrt-2* mutants defective for the DNA damage checkpoint and *in vivo* telomerase activity grown for generations exhibited increased genomic rearrangements with some evidence of chromothripsis [2]. MRT-2 is the *C. elegans* RAD1 subunit of the conserved 9-1-1 DNA damage checkpoint complex. *mrt-2* mutants are defective for the DNA damage checkpoint and *in vivo* telomerase activity; telomeres shorten progressively over generations until the population succumbs to sterility [32]. Previous studies described the isolation and initial characterization of stable chromosome fusions from late generation animals indicating that once telomeres become critically short, chromosomes engage in end-to-end fusions [32]. Such chromosomal fusions associated with telomere shortening, using array technology to assess copy number changes, were linked to fusions that involve replication fork stalling and template switching processes leading to replication-induced duplication processes close to the fusion points [33]. Our studies based on NGS provided examples where two chromosomes with critically short telomeric sequences undergo a series of breakage fusion bridge (BFB) cycles followed by a joining event that bears resemblance to chromothripsis [2]. During BFB cycles, first postulated by Barbara McClintock in maize, two sister chromatids fuse in G2 and are ripped apart close to their terminals during cell division. Such broken chromosomes undergo further cycles of fusion and breakage [34,35]. The pattern of genomic rearrangements seen in *mrt-2* mutants can be explained by several such BFB cycles, followed by an event involving multiple breaks and joins, concomitant with the generation of the sequenced fusion chromosome (Fig. 2B). This final step involving the random insertion of chromosomal fragments between the two fused chromosomes is akin to chromothripsis, where large numbers of chromosomal fragments are randomly assembled [10]. Intriguingly, a recent study on fusion chromosomes in lymphoblastic leukemia similarly suggests the presence of initial rounds of BFB cycles prior to chromosome-to-chromosome fusions with interstitial insertions of chromosome fragments [36]. Therefore, the analysis of *C. elegans* mutants with progressive telomere shortening combined with cancer mutation profiles support a contribution of telomere attrition to chromothripsis etiology.

A second mutational signature associated with large genomic rearrangements is chromoanasynthesis (“chromo” for chromosomes and “anasynthesis” for reconstitution), a signature predominantly seen in inherited constitutional genomic disorders. Chromoanasynthesis is characterized by localized copy number changes involving DNA sequences from near-by genomic regions with flanking homology. It likely arises when locally impaired DNA replication initiates serial microhomology-driven invasions of nearby genomic regions, often crossing back and forth between regions, thus converging in chromosomal rearrangement and copy number gains. Interestingly, exposure of different *C. elegans* mutant genotypes to cisplatin and mechlorethamine, chemotherapy drugs used in the treatment of a broad range of cancers such as sarcoma, lymphoma, germ cell tumors revealed genomic rearrangements with features of resembling chromoanasynthesis [2]. Several lines of evidence suggest that these events are linked to persistent DNA cross-links. Chromoanasynthesis occurs with both chemically distinct DNA cross-linking agents, while both agents at the level of single nucleotide changes lead to distinct signatures, likely associated with monoadducts. Furthermore, these chromoanasynthesis-like rearrangements occur in genomic DNA derived from the clonal progeny of a zygote formed from single, transiently exposed oocytes and sperm cells (Fig. 1B). Thus, each cluster, resulting from excessive mis-templated, localized hyper-replication must have occurred in a single catastrophic event. Several DNA repair pathways, including the Fanconi Anemia pathway, double-strand break repair and translesion synthesis are needed for DNA cross-link repair. Consistent with this, the number and severity of chromoanasynthesis-like clustered rearrangements was increased in Fanconi Anemia mutants as well as in *xpf-1*, which has been discussed to be directly involved in cross-link repair [37–39]. Such chromoanasynthesis-like events also occurred in mutants defective in homologous recombination, a repair modality needed to meld DNA double-strand breaks generated during DNA cross-link repair. Observing the most severe cases of chromoanasynthesis in *xpf-1* mutants is in line with a reported overexpression of ERCC1/XPF in cisplatin-resistant cancers. Interestingly, the analysis of cisplatin-induced mutagenesis in various genetic backgrounds indicates that repair factors, normally associated with homologous recombination like SLX-1 and MUS-81 can lead to reduced mutagenesis. Small deletions created by cisplatin are less frequent in *slx-1* and *mus-81* mutants [2]. Intriguingly it also appears that structural rearrangements arise less frequently in DNA end-joining mutants, in line with earlier reports that this repair modality might illegitimately join broken chromosomes which form as intermediates of DNA cross-link repair [40,41]. In summary, NGS sequencing of *C. elegans* treated with cisplatin and analyzed across many DNA repair deficient backgrounds allows to reconstruct mutation signatures associated with complex chromosomal rearrangements in patients. Furthermore, the role of individual repair factors can be deduced.

## Future directions

New technological developments involving NGS allow for an understanding of mutational processes at a genome-wide level and complement classical approaches used for studying DNA repair processes. For instance, the analysis of DNA breakpoints in *dog-1* and *polh-1*; *polk-1* mutants identified an imprint of the DNA repair mechanism involved in the generation of deletions and a subsequent identification of the role of *polq-1*. Furthermore, using next generation sequencing, the etiology of complex genetic rearrangements occurring in hereditary disease and cancer cells could be mimicked in *C. elegans*, providing insight into the relevant mutagenic mechanisms. The systematic analysis of mutagenesis, occurring in wild-type and DNA repair deficient backgrounds, with and without DNA damaging agents, will allow for reconstituting mutational signatures emerging from the analysis of cancer genomes. Furthermore, important insights into DNA repair mechanisms, especially those, which are inherently error prone, will be possible.

## Figures and Tables

**Fig. 1:**
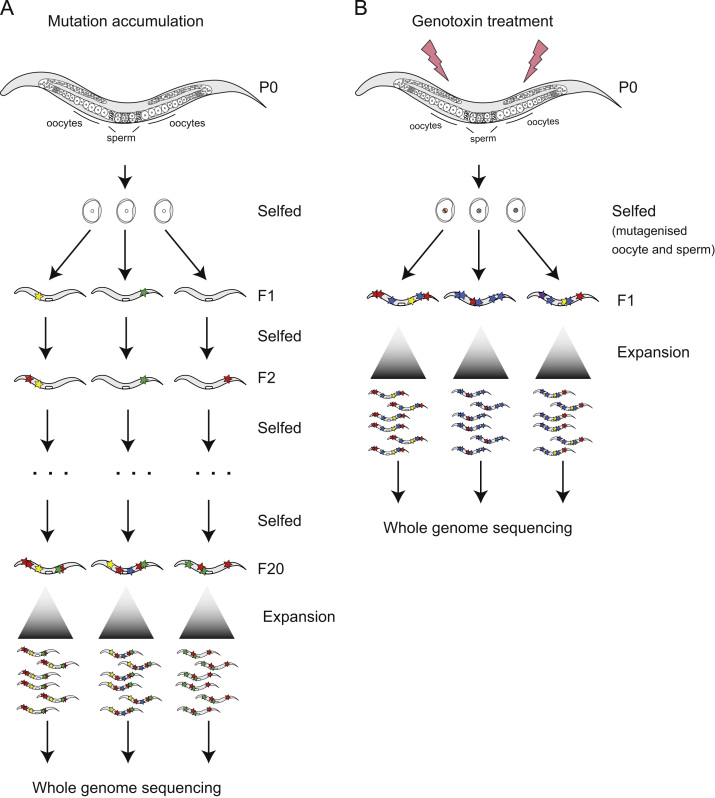
Schematics of mutation accumulation in *C. elegans* grown over generations or following exposure to genotoxic agents. (A) Several individual progeny of a parental (P0) worm are propagated by self-fertilization for 20 generations, randomly choosing single L4 larvae each line and generation (F1=filial generation 1, F20=filial generation 20). The F20 worm from each line is expanded to generate sufficient DNA for whole genome sequencing. Mutations (indicated by colored stars) arising at any given generation have a one in four chance to manifest as homozygous in the following generation. (B) Individual P0 worms are treated with different doses of genotoxin and allowed to self-fertilize. Three F1 worms, each bearing a number of heterozygous mutations, are expanded to produce sufficient DNA for whole genome sequencing.

**Fig. 2:**
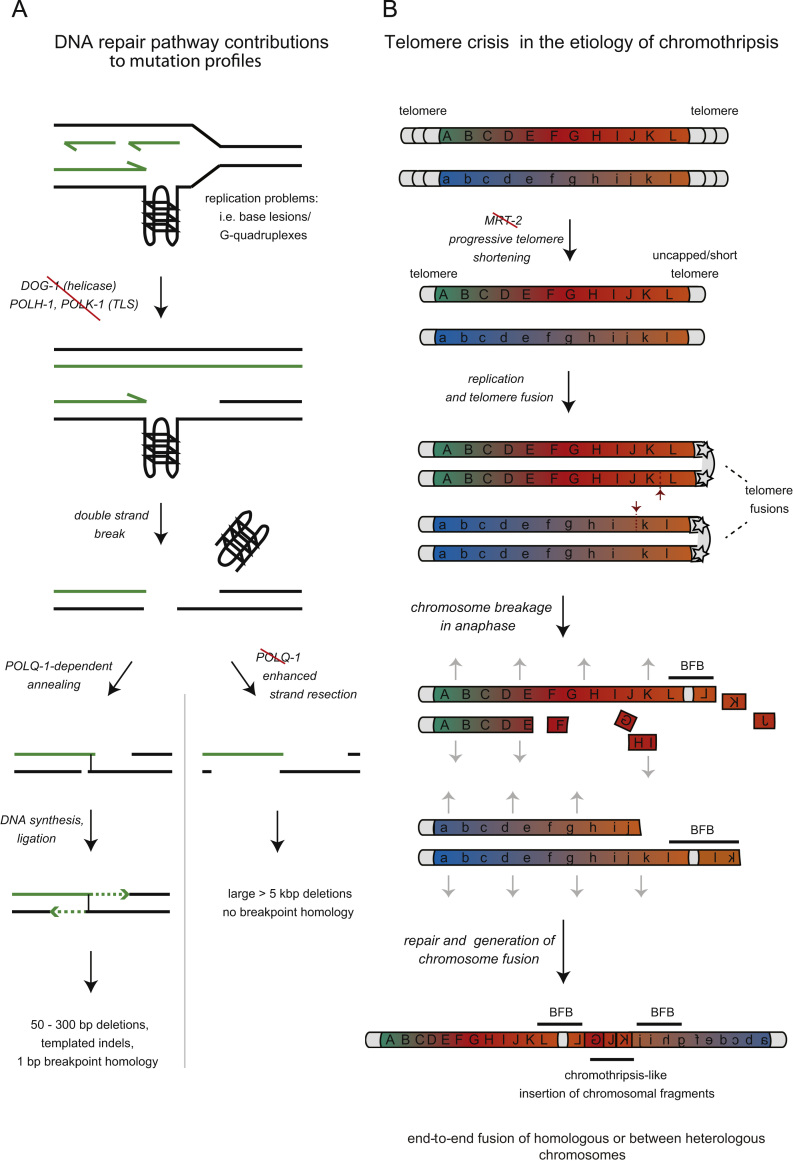
Schematics of DNA repair pathway choice in the repair of G-quadruplex structures and a possible mechanism for the generation of chromothripsis-like events during telomere attrition. (A) DNA repair pathway choice in the repair of G-quadruplex structures during replication. Green lines indicate newly synthesized DNA; blue lines represent a homologous DNA sequence. (B) Generation of chromothripsis-like rearrangements following telomere crisis. Sister chromatid fusions occur at critically short telomeres. Such fusion chromosomes are ripped apart during the following cell division (red arrows) leading to the gain or loss of chromosome terminal sequences. Chromosomes that have undergone one or more such breakage-fusion-bridge (BFB) cycles fuse integrating chromosomal fragments randomly at the fusion sites (K, J, and G in lowest panel), leading to chromothripsis-like mutation signatures. Gray arrows represent the direction of pulling forces on sister chromatids during anaphase.
